# Neurodevelopmental outcomes of moderately preterm birth: precursors of attention deficit hyperactivity disorder at preschool age

**DOI:** 10.1186/2193-1801-2-221

**Published:** 2013-05-12

**Authors:** Giovanna Perricone, M Regina Morales, Germana Anzalone

**Affiliations:** Department of Psychology, University of Palermo, Italy, Viale delle Scienze – Ed. 15, Palermo, 90128 Italy; Faculty of Educational Sciences, University of Palermo, Italy, Viale delle Scienze – Ed. 15, Palermo, 90128 Italy

**Keywords:** Attention deficit hyperactivity disorders’ risk, Precursors, Moderately preterm, Preschool age

## Abstract

Moderately preterm birth seems to be an evolutional risk condition at cognitive, behavioural and socio-relational levels. The study is aimed to investigate the likely occurrence of precursors of Attention Deficit Hyperactivity Disorder (ADHD) in moderately preterm children at preschool age.

The research involved an experimental group made up of 50 moderately preterm children (mean: 34.6 weeks’ gestational age, standard deviation [SD]: 2) without any medical and neurologic neonatal complications and low birth weight (mean:2100g., SD: 350g.) and a check group of 50 full term born children. Parents and teachers of children were administered specific questionnaires to detect ADHD.

The outcomes show a risk of ADHD highlighting statically significant differences related to gender [F_(2, 99)_ = 2.99, p = .04], birth [F_(2, 99)_ = 9.6, p = .03] and interaction [F_(2, 99)_ = 2.2, p = .01]. The moderately preterm children showed deficit in self-regulation [F_(2, 99)_ = 1.14, p = .04] and attention deficit in daily life both in family [F_(2, 99)_ = 7.8, p = .04] and school contexts [F_(1, 99)_ = 3.3, p = .04].

The outcomes hint assessment paths aimed to monitor the aspects of cognitive, motor, behavioural development of moderately preterm children recognised as signs of problematic functioning profiles. Therefore, specific training will have been designed since preschool age in order to control the ADHD risk factors.

## Background

Moderately preterm birth (Gestational Age - GA - between 32 and 34/5 weeks), especially when occurs together with low birth weight (< 2500 g.), seems to be an evolutional risk condition at cognitive, behavioural and socio-relational levels (Cherkes-Julkowski, 1998; Huddy et al., 2001; Bhutta et al., 2002; McGrath and Sullivan, 2002; Aylward, 2005). Some studies reported that the interruption of the foetal maturation – occurring at 32nd and 36th gestational week, leads to cerebral immaturity at birth (Inder et al., 2005; van Baar et al., 2009; Heinonen et al., 2010) and it represents a risk for cognitive development of preterm children (Peterson et al., 2003; Roccella et al., 2004; Kinney, 2006; Krain and Castellanos, 2006; Espy et al., 2007; Parisi et al., 2007). The attentional, metacognitive, self-regulation impairments may lead in turn the development of Attention Deficit And Hyperactivity Disorder (ADHD) (Chyi et al., 2008).

Moreover, such studies suggest that also moderately preterm children (GA < 32 weeks), show general self-control and attentional disorders since preschool age, though at a less severe level as compared with severely preterm cases (Huddy et al., 2001; van Baar et al., 2009; Heinonen et al., 2010; Perricone and Morales, 2011a; Perricone et al. 2012a). These disorders are not detected till school age, when the combination of symptoms is more easily diagnosed (Perricone et al. 2012b; Huddy et al., 2001; van Baar et al., 2009; Heinonen et al., 2010). Indeed, cognitive disorders in this period can be revealed by symptomatic disadaptive behaviour in child reference educational contexts such as family, group of peers, etc. and especially at school, since it is characterized by specific time and spaces, organized by a discipline referring to metacognitive process and the use of diversified languages and multiple modalities of elaboration of information (Perricone and Morales, 2011b; Perricone et al. 2012aPerricone et al. 2012c).

This study differs from other research projects studying pregnancy or birth complications in the etiology and pathogenesis of Attention Deficit And Hyperactivity Disorder (ADHD) (Hartsough and Lambert, 1985; Lou, 1996). They have explored the biological influences of the development of emotional and behavioural regulation in extremely preterm children. Preterm children as well as children who have markedly lower birth weights are at high risk for later inattention, hyperactivity or Attention Deficit And Hyperactivity Disorder at school age (Szatmari et al., 1993; Breslau et al., 1996; Pinto-Martin et al., 2004). The innovative aspects of this study are: focus on moderately preterm children as well as on the precursors of Attention Deficit And Hyperactivity Disorder at preschool age.

This research project is aimed at detecting the presence of precursors of Attention Deficit And Hyperactivity Disorder (ADHD), in a group of Italian moderately preterm children at preschool age, characterized by distraction, impulsiveness and hyperactivity. The reason is the average onset of Attention Deficit And Hyperactivity Disorder (ADHD) symptoms, as noted earlier, is often displayed at preschool age - 3 to 4-year-old children and, more generally, on their entry into formal schooling. Preschool-aged children, who are perceived as difficult and resistant to control, or who display inattentive and hyperactive behaviour that persists for at least a year or more, are highly likely to have Attention Deficit And Hyperactivity Disorder (ADHD) and to remain so into elementary school years (American Psychiatric Association - APA, 1994) and even adolescence (Byrne et al., 2000; Daley, 2006; Curatolo et al. 2010).

Identification of such precursors could increase knowledge on some specific features of the evolutional paths in order to provide directions for the activation of training procedures to improve the quality of life of moderately preterm children (Perricone and Morales, 2011a).

The study deals with particular specific indicators of the precursors of Attention Deficit And Hyperactivity Disorder (ADHD), identified as disattention/inattention meant as distraction during school and recreational activities and in following instructions to fulfil an activity. A wide literature has highlighted that preterm children are less capable to focus attention on objects or activities (Gillberg, 2003; Lawson and Ruff, 2004; McGrath et al., 2005; Espy et al., 2007; Shum et al., 2008) as well as to activate cognitive processes (gathering and selecting information, concentration…) functional for a more rational adjustment to situations (Byrne et al., 2000; Daley, 2006; Curatolo et al. 2010).

Among these indicators, hyperactivity includes restlessness, difficulties in “calm” play activities and motor activity modulation disorder leading to perform clumsy and uncoordinated movements with a reckless motor behaviour (Foulder-Hughes and Cooke, 2003; Hemgren and Persson, 2006; Clark et al., 2008). Indeed, in extremely preterm children, some motor development impairments occur especially with regard to the capacity of modulation of fine motor skills and visuospatial coordination (Hemgren and Persson, 2006; Clark et al., 2008). One more indicator, linked to hyperactivity is impulsivity, expressing as impairment in behavioural self-regulation, motor skills and self-control. Due to impulsivity, the child has difficulty in respecting the roles, waiting for his/her turn, modulating his/her relationship with the other, intervening in and “invading” others’ activities (plays, conservations, etc.), waiting before speaking and has incapability of staying still in long waits and postponing gratifications (Befera and Barkley, 1985). The reading model, according to the literature of the field, recognizes that males are likely to be more affected by the precursors of Attention Deficit And Hyperactivity Disorder (ADHD) than females (Befera and Barkley, 1985; Lawson and Ruff, 2004) especially with respect to hyperactivity.

## Methods

### Objectives and hypothesis of the research

The aim of the study is to investigate the presence or lack, of precursors of Attention Deficit And Hyperactivity Disorder (ADHD) in moderately preterm children at preschool age. It wants to verify the presence of statistically significative differences among precursors of Attention Deficit And Hyperactivity Disorder between a group of moderately preterm children and group of full term children.

### Participants

- 50 moderately preterm children (mean: 34.6 weeks’ gestational age, standard deviation [SD]: 2) without any medical neonatal and/or complications and low birth weight (mean: 2100g., SD: 350 g.) (Table 1), were selected according to the following criteria: gestational age <35 weeks, birth weight 1500 to 2500 g. without any neurologic pathology, sensorial and genetic pathology deficit nor malformative syndrome.
- 50 healthy full term children (mean: 40 weeks’ gestational age, without any pre and perinatal complications), comparable to the patient’s group for socio cultural features were also recruited (Table 1). The selection criteria of control group were: birth at about 40^th^ postconceptional week (range = 39 to 41 weeks of gestation); birth weight > 2500 g.; absence of pre and perinatal complications, neurologic pathology, sensorial and genetic pathology deficit and malformative syndrome.

**Table 1:** **Prenatal and family characteristics of the sample by term and preterm birth**

Characteristics of children				Characteristics of children full term (=50)		
Moderately preterm* (=50)						
***Variable***	*mean*	*sd*	*range*	*mean*	*sd*	*range*
Child age (months)	62	4	57-67	64	2.5	61-66
Birth Gestational Age	34,6	2	31–35	40	2.5	38–41
Birth Weight (g)	2100	350	1750-2450	3200	200	3000-3400
Days of Hospitalization	15	8	8–23	2	1.5	2–3
Female %	54%				51%	
Male %	46%				49%	
**Family background of children born preterm**				**Family background of children born full term**		
***Variable***	*mean*	*sd*	*range*	*mean*	*sd*	*range*
Mother’s age (years)	30,6	6	24-37	32,6	5	28-38
Parental educational attainment (years)	13	8	8-23	12	8	8-22
Number of children	2	1.5	1-3	2	1.5	1-4

* Healthy Preterm (HPT): No medical/neurological complications.

All the involved children had an average age of 5.2 years and attended the last year of Italian preschool (namely the English equivalent for the first year of primary school). Furthermore, they did not show any cognitive deficit, nor clinically significant learning disorders as tested with a preliminary assessment of specific cognitive skills (metacognition, attention, memory, orientation).

Before involving the research group children, the official authorities had approved the proposed path in terms of correctness and ethics. Then the children's parents were asked to sign the declaration of informed consent according to the D.LGS. 196/2003 art.13 related to their personal and other people’s data protection. Professionals working in the field have followed the official path respecting the criteria that regulate the main codes of ethics of the study and in compliance with the Helsinki Declaration.

The research group children were involved after their parents had signed the declaration of informed consent according to the D.LGS. 196/2003 art.13 related to the personal data protection.

The subjects constituting the two groups were selected by means of a survey on birth carried out in classroom. The children, together with their parents, were asked to answer some simple questions about their birth (“I was born on….; I was born in…..; I was/not born prematurely….; my birth weight was…; I was hospitalized for ….”). After having compared the information regarding the medical and birth records of children, which the parents had provided the researchers with, the children were divided into two groups (experimental group and control groups), and the research was carried out.

The children involved were all born in Palermo and province and the preterm infants were admitted to Neonatal Intensive Care Unit (NICU) of three hospitals in the Sicilian region.

In relation to the family’s socioeconomic status and the socio-family background of the research group (Table 1), the children of the two groups (group of children moderately preterm and group of children full term) do not differ in a significative way and are therefore interchangeable.

To be more precise, the full term and preterm children belong to Italian families, 90% of them have siblings (on average two children per family), whose mother (on average 32 years old) belongs to a social middle class (one-income family), on average with secondary school education.

### Procedures and instruments

The following instruments were used:

- IPDDAI Italian scale (*Attention Deficit Hyperactivity Disorder Early Detection for Teachers*) (Marcotto, Paltenghi and Cornoldi, Marcotto et al. 2002): a specific questionnaire, articulated in 18 items that allows to investigate distraction and hyperactivities symptoms in children 5 to 6 years old, the last year of Italian pre-school (namely the English equivalent for the first year of primary school). The children’s reference teachers filled in the questionnaire. They were asked to evaluate how frequently they detected certain skills and behaviours showed by children in classroom, by the means of Likert-type Scale 4 response levels (0 = not at all/never; 1 = rarely/sometimes; 2 = quite frequently/most of times; very frequently/always). Teachers were blind as to the variable prematurity of the tested children. The 14 items of the questionnaire refer to the dimensions of *disattention*, i.e. the difficulty the child has in focusing on details and prolong his/her attention during an activity and of *hyperactivity/impulsivity*, i.e. motor skills restlessness, self-control and self-regulation disorders. The last four items are related to the likely risk factors that may affect the persistency, grade and development of ADHD symptoms such as socio-cultural disadvantage, poor cognitive potentialities, presence of emotional and/or relational impairments and likely family troubles. The analysis of data allows to distinguish scores related to each subscale (*disattention* and *hyperactivity/impulsivity*). More specifically, a score equal to or lower than 10^th^ percentile (raw score equal to or higher than 14 points per dimension) identifies a case at risk; a score between 11^th^ and 24^th^ percentile (score equal to or higher than 9) identifies an edge case; a score equal to or higher than 25^th^ percentile identifies a non problematic case.
  The instrument has been designed according to Italian normative sample (Marcotto et al. 2002). It has been performed a factorial analysis on the items that refer to diagnostic criteria of DSM-IV and Kendall and Wilcox’s Scale. Such analysis allowed to assess good levels of saturation of each item with respect to the two dimensions that the instrument needs to evaluate. The predictive quality of the instrument is very good.
- IPDDAG Italian Scale (*Attention Deficit Hyperactivity Disorder Early Detection for Parents*) (Riello et al. 2005): a specific questionnaire aimed to the detection of subjects defined “at risk” of ADHD during (pre-)school age, performed through the assessment of parents. In particular the questionnaire has been administered to parents of children attending the last year of Italian pre-school (namely the English equivalent for the first year of primary school). Parents were asked to evaluate through the Likert-type scale 4 response levels (0 = not at all/never; 1 = rarely/sometimes; 2 = quite frequently/most of the time; very frequently/always) how frequently they had observed the behaviour their children have at home. The questionnaire is made up of 14 items expressed in a negative form, whose odd items refer to disattention, related to the difficulty the child has in prolonging his/her attention on an activity and plays at home, while the evens refer to *impulsivity/hyperactivity* meant as motor restlessness and self-control and self-regulation disorders. Five more final items are also to be considered related to risk factors (forms of socio-cultural disadvantage, poor cognitive potentialities, presence of emotional and/or relational impairments and likely family troubles). The content of items refers to other previous questionnaires, namely IPDDAI scale and SDAG scale (addressed to parents in order to assess disattention and hyperactivity behaviour of children at school age) and DSM-IV criteria. The validity of the scale has been verified through a factorial analysis whose results show that the division of items in components (disattention and hyperactivity) have been realized (Riello et al. 2005). Moreover, the values of Pearson’s coefficients of correlation and Cronbach’s alpha value showed a good internal coherence of the instrument.
  The higher the score, the larger the presence of patognomonic tracts of the disorder is. Therefore, a score equal to or higher than 14 marks in each scale identifies a subject at risk.

### Data treatment and analysis

The statistic programme for Social Sciences – SPSS 16 was used to analyze data.

Kolmogorov-Smirnov’s test was used to verify the presence of a normal distribution (IPDDAI e IPDDAG p > .05) and Levene’s test was used to verify the equality of variances in the two samples (homogeneity of variance) (p > .05). Then, a multi-varied covariance (MANCOVA) with continuous variables (scores related to the different scales) 2 (birth) × 2 (gender) was used. More specifically, the likely differences between the scores of the various scales (dependent variables) have been analyzed considering birth (Term/Preterm) and gender (F/M) as independent variables and the birth weight as covariate variable. A value of p = 0.05 was considered significant.

## Results

As to the assessments by teachers (IPDDAI) and parents (IPDDAG), the results showed significant effects of gender (IPDDAI, [F_(2, 99)_ = 2.99, p = .04, η^2^ = .32]) and birth (IPDDAG, [F_(2, 99)_ = 9.6, p = .038, η^2^ = .11]), and interaction gender X birth (IPDDAI, [F_(2, 99)_ = 2.2, p = .01, η^2^ = .24]). There were not statistically significant effects related to the variable “birth weight” (Table 2).

**Table 2:** **Precursors of attention deficit and hyperactivity/impulsivity in moderately preterm and full term children**

Mothers - IPDDAG Questionnarie									
*Scales*	*MPT*			*T*			*Tests of Between-Subjects Effects*		
	*Mean (sd)*	*Mean Female (sd)*	*Mean Male (sd)*	*Mean (sd)*	*Mean Female (sd)*	*Mean Male (sd)*	*Birth (MPT/T)*	*Sex (M/F)*	Sex * Birth
Disattention	10.01	10.7	9.8	8	7	9	F _(df1,99)_ 7.8 p .04	F _(df1, 99)_ .34 p .5	F _(df1, 99)_ 2,5 p .12
	(5.9)	(5.9)	(5.9)	(5.3)	(4.7)	(5.9)			
Hyperactivity/ Impulsivity	15.67	15.6	15.2	13.5	13	14.4	F _(df1, 99)_ 1.1 p .04	F _(df1, 99)_ 07 p .8	F _(df1, 99)_ 4 p .52
	(5.1)	(6.6)	(6.7)	(6.5)	(6.3)	(6.6)			
Teachers - IPDDAI Questionnarie									
*Scales*	*MPT*			*T*			*Tests of Between-Subjects Effects*		
	*Mean (SD)*	*Mean Female (SD)*	*Mean Male (SD)*	*Mean (SD)*	*Mean Female (SD)*	*Mean Male (SD)*	*Birth (MPT/T)*	*Sex (M/F)*	Sex * Birth
Disattention	9.3	10	8.3	5.6	5.1	6.1	F _(df1, 99)_ .45 p .5	F _(df1, 99)_ .16 p .69	F _(df1, 99)_ 3.3 p .04
	(5.2)	(4.8)	(4.8)	(4.9)	(4.8)	(5)			
Hyperactivity/ Impulsivity	13.04	13.3	13.1	5.3	4.6	6.1	F _(df1, 99)_ .86 p .35	F _(df1, 99)._ 52 p .47	F _(df1, 99)_ 1.02 p .31
	(5.3)	(5.3)	(5.4)	(5.2)	(5.1)	(5.2)			

*Legend:* MPT: Moderately Preterm; T: Term children; sd: Std. Deviation; IPDDAG: Attention Deficit Hyperactivity Disorder Early Detection for Parents; IPDDAI: Attention Deficit Hyperactivity Disorder Early Detection for Teachers.

So in parents’ evaluation (IPDDAG), moderately preterm and full term children differ with relation to the presence of ADHD precursors, while in teachers’ assessment such difference seems to occur both with relation to the “gender” variable and jointly to “birth” variable. This indicates that moderately preterm little boys/girls compared to full term children have different attention deficits and behavioural self-regulation disorders.

In the teachers’ assessment through the IPDDAI, there was a significant interaction between gender and birth in *disattention* [F_(1, 99)_ = 3.3, p = .04, η^2^ = .005]. Preterm little girls, as highlighted by the mean scores of the research group, have higher scores in comparison with preterm and full term little boys and girls (Table 2).

It seems that preterm little girls mostly show difficulty in prolonging attention on their homework and preschool activities, so they are often distracted, they try to avoid the activities requiring mental effort or attention to details, or the organization of activities as well.

Such datum is also confirmed by information given by parents (IPDDAG) that showed statistically significant differences related to preterm birth in both *hyperactivity/impulsivity* [F_(1, 99)_ = 1.14, p = .046, η^2^ = .02] and *disattention* [F_(1, 99)_ = 7.8, p = .048, η^2^ = .014], where preterm children get higher scores in these two dimensions compared to full term children (Table 2). So, parents gave a description of moderately preterm children’ behaviour as children who, regardless of their gender, not only show attention deficit at home concurring with the evaluation of behaviour in classroom provided by teachers, but also show restlessness, excessive and restless movements, difficulties in modulating motor skills activities with clumsy incoordinated reckless and not aimed movements.

In relation to such data, it has to be considered the relevance of scores of preterm children with regard to hyperactivity and disattention precursors since they are lower than the cut off score fixed in the questionnaire. Specifically, considering the cases when parents and teacher agree (Cohen’s agreement inter rater reliability k index has been calculated and has highlighted acceptable levels of agreement – = .78), the 30% of moderately preterm children (n. 15) exceeds the cut-off score being at risk of attention deficit, and 14% of them is on the edge (n. 7); only the 14% of full term children (n. 7) seems to be at risk and the 6% of them is on the edge (n. 3). A greater number of children moderately preterm born, compared with children full term born, are at risk of precursors of inattention (χ2 = 6.6, gf = 2, p = .03).

With regards to hyperactivity, the 54% of preterm children seems to be at risk (n. 27) and 14% is on the edge (n. 7); 20% of full term children are at risk (n. 10) and 6% of them is on the edge (n. 3). Even in this case, the number of children moderately preterm born characterized by precursors of hyperactivity, appears statistically higher than in the group of children full term born (χ2 = 17.7, gf = 2, p = .001).

## Conclusion

The main results of the study highlight the presence of a profile of moderately preterm children who, even at preschool age, are “at risk” of precursors of attention deficit and hyperactivity disorder. Preterm children of the research group are described, especially at home, as hyperactive and restless, children, showing difficulties in self-regulating and self-controlling during calm play activities. These data seem to be significant especially when they concern the family contexts of the preterm infants involved who do not appear to be at risk. The family’s socioeconomic status and the socio-family background of the research group was high. However, it should be noted that it was not analyzed the contribution of social contextual factors such as the availability of socioeconomic resources, maternal psychological well-being, and parenting. This contribution will be better explored by further research projects.

A further remarkable element underlined by data and related to disattention, is that parents and teachers agree in reporting that moderately preterm children show, both at school and home, attention difficulties that are at risk of attention deficit at preschool age. This aspect seems strongly characterized in terms of gender typization: both parents and teachers recognize attention’s difficulty in moderately preterm children, even if teachers detect a higher impairment in females (see Table 2).

Such data trace a similarity between the maturative and developmental path of severely preterm children (< 32 weeks) and that of moderately preterm ones (van Baar et al., 2009) highlighting, even in the latter case, likely risks linked to moderately preterm birth. Such data are in accord with those studies that have highlighted that severe prematurity and low birth weight, occurring together or separately, are factors that may affect (van Baar et al., 2009; Strang-Karlsson et al., 2008) the development of impulsivity regulation, self-regulation and behaviour and motor self-control processes (as well as concentration and attention skills). Prematurity and low birth weight (aside from gestational age of foetus) are important factors that lead to underline that children of the research group are characterized by a moderately prematurity (32-34 weeks) without any neurologic sequence, cognitive impairments, prolonged hospitalizations in NICU (on the average 15 days) nor surgical interventions, but a low birth weight (mean =2100 g.). The levels of maturity of physiological brain processes of foetus reached before their interruption caused by birth at 34 weeks (van Baar et al., 2009) are important too, because together with genetic and environmental factors (early interaction with hospital environment, medical treatments, relationships with parents, external stimuli…), contribute to trace problems and slight disorders only occurring in terms of “slowness” and/or difficulty at preschool and school age.

However, the use of a relatively small-hospital-based sample of NICU children could be the limit of such a study. Children who had no complaints after neonatal intensive care, those who were being treated in rehabilitation centres because of severe disabilities, and children who were followed-up in other hospitals were not included in the sample. This may have caused both underestimation and overestimation of the precursors of ADHD.

Finally, an analysis of both clinical and socio-family factors will be important in developing a better understanding of the processes shaping the developmental pathways toward regulatory competence in children born preterm.

In conclusion, the present study provides some hints for prevention of ADHD risk in moderately preterm children. Early training courses addressed to preschool children would help them to develop self-regulation skills (emotional, cognitive, relational, behavioural) (Perricone Briulotta, 2012; Perricone et al. 2012a); when addressed to parents they would help to develop their parental competencies in order to face their child behavioural problems related to everyday life; specific preschool and school educational paths addressed to teachers would help develop didactical strategies oriented to the development of cognitive and meta-cognitive competencies in children.

A further implication of the study is related to the likely spin off of the results in paediatric-health field. Indeed the study suggests the need of hypothesizing, not only in the case of severe prematurity, specific paths of assessment of the various evolutional dimensions of children aimed to monitor certain aspects of their cognitive, motor, socio-relational, behavioural development, etc… They are paths following the perspective of Paediatric Psychology (Perricone Briulotta, 2012; Roberts et al. 1988; Spirito et al., 2003) that follow the neonatal follow-ups performed by hospital medical staff and health assessments performed by paediatrics and that allow a more complex “reading” of the evolutional profile of moderately preterm children.

## Consent

The research group children were involved after their parents had signed the declaration of informed consent according to the D.LGS. 196/2003 art.13 related to their personal and other people’s data protection. Written informed consent was obtained from the patient’s parent for publication of this report.
